# Measurement of Protein Mobility in *Listeria monocytogenes* Reveals a Unique Tolerance to Osmotic Stress and Temperature Dependence of Diffusion

**DOI:** 10.3389/fmicb.2021.640149

**Published:** 2021-02-17

**Authors:** Buu Minh Tran, Haritha Prabha, Aditya Iyer, Conor O’Byrne, Tjakko Abee, Bert Poolman

**Affiliations:** ^1^Department of Biochemistry, University of Groningen, Groningen, Netherlands; ^2^School of Natural Sciences, National University of Ireland, Galway, Ireland; ^3^Laboratory of Food Microbiology, Wageningen University Research, Wageningen, Netherlands

**Keywords:** protein mobility, lateral diffusion, *Listeria monocytogenes*, osmotic stress, fluorescence recovery after photobleaching, macromolecular crowding, stress response

## Abstract

Protein mobility in the cytoplasm is essential for cellular functions, and slow diffusion may limit the rates of biochemical reactions in the living cell. Here, we determined the apparent lateral diffusion coefficient (*D*_*L*_) of GFP in *Listeria monocytogenes* as a function of osmotic stress, temperature, and media composition. We find that *D*_*L*_ is much less affected by hyperosmotic stress in *L. monocytogenes* than under similar conditions in *Lactococcus lactis* and *Escherichia coli*. We find a temperature optimum for protein diffusion in *L. monocytogenes* at 30°C, which deviates from predicted trends from the generalized Stokes-Einstein equation under dilute conditions and suggests that the structure of the cytoplasm and macromolecular crowding vary as a function of temperature. The turgor pressure of *L. monocytogenes* is comparable to other Gram-positive bacteria like *Bacillus subtilis* and *L. lactis* but higher in a knockout strain lacking the stress-inducible sigma factor SigB. We discuss these findings in the context of how *L. monocytogenes* survives during environmental transmission and interaction with the human host.

## Introduction

A universal property of life is that the components in a cell move around, and in bacteria, this motion is diffusive and not driven by metabolic energy. In an aqueous solution, the mobility or lateral diffusion coefficient (*D*_*L*_) of globular proteins is given by the Stokes-Einstein relation, and in living cells, *D*_*L*_ values are lower because of a multitude of effects not limited to macromolecular crowding, phase separation, and compartmentalization (Konopka et al., 2009; Mika and Poolman, 2011; Parry et al., 2014; Joyner et al., 2016; Munder et al., 2016; Schavemaker et al., 2018). Knowing the *D*_*L*_ of proteins, their possible confinement, and transient interactions are essential for a quantitative description of the biochemical processes in the cell.

The macromolecular crowdedness in the cell is conspicuous but still underappreciated (Ellis, 2001; Rivas and Minton, 2016; van den Berg et al., 2017). The inside of cells (and cell membranes) is filled with a high density of macromolecules. The concentration of cytoplasmic protein, RNA, and DNA range from 200–320 mg/ml, 75–120 mg/ml, and 11–18 mg/ml, respectively, that is, for *Escherichia coli* cells grown in media of osmolality varying from 0.1 to 1.02 Osm (Cayley et al., 1991). Although *E. coli* is by far best studied, similar numbers have been reported for other bacteria, and a crowding wherein the macromolecules occupy a volume of 15 to 20% of the cytosol may be generally valid for all forms of life (Zimmerman and Trach, 1991; Cayley and Record, 2003, 2004; Boersma et al., 2015; van den Berg et al., 2017). The excluded volume of the macromolecules influences the activities of cytoplasmic molecules, favors in general self-association of proteins, and impacts the condensation of nucleic acids (de Vries, 2010; Kim et al., 2011). Furthermore, the high excluded volume can influence reaction rates in the cell (Schavemaker et al., 2018).

At an ambient temperature of 298K (24.85°C) and in an aqueous solution, GFP has *D*_*L*_ ∼87 μm^2^/s, which according to the Stokes-Einstein’s equation matches a globular particle with a Stokes radius of 2.82 nm (Miller, 1924; Terry et al., 1995; Swaminathan et al., 1997). The *D*_*L*_ values of GFP in the cytosol vary from 20 to 30 μm^2^/s in Chinese hamster ovary (CHO) and *Dictyostelium* amoebae cells (Swaminathan et al., 1997; Potma et al., 2001; Verkman, 2002) to 3–14 μm^2^/s in bacterial cells (Elowitz et al., 1999; Konopka et al., 2009; Mika and Poolman, 2011; Mika et al., 2014), indicative of increased macromolecular crowding in prokaryotic compared to eukaryotic cells. Apart from macromolecular crowding, the lateral diffusion of macromolecules is also influenced by surface properties of the molecules; cationic proteins interact with the ribosomes, which slows down their diffusion both in prokaryotic (Schavemaker et al., 2017) and in mammalian cells (Xiang et al., 2020).

The mobility of proteins in the cell depends on the osmolality of the growth medium, and *D*_*L*_ in the cytoplasm decreases upon osmotic upshift (hypertonicity) and can increase under hypotonic conditions. For instance, *D*_*L*_ of GFP in the cytosol of the amoebae increases twofold (46 μm^2^/s) when cells are suspended in distilled water (hypoosmotic) and decrease to 17 μm^2^/s in 300 mM sorbitol solution (Potma et al., 2001). Similar trends are seen in other eukaryotic cells (Swaminathan et al., 1997; Xiang et al., 2020). The *D*_*L*_ of GFP in *E. coli* drops from 14 to 6 μm^2^/s when the medium osmolality is increased from 0.28 to 1.45 Osm, and contrary to the observations in eukaryotic cells, the *D*_*L*_ also decreases when cells are subjected to hypotonic conditions (0.1 Osm) (Konopka et al., 2009). We emphasize that the effect of medium osmolality is attenuated in cells with a high turgor, as initially the osmotic pressure difference across the membrane will be affected, which can be without much effect on the cell volume and thus the internal crowding. The turgor is small in cells without a wall but can vary from a few atmospheres in Gram-negative bacteria to more than 10 atm in Gram-positive bacteria (Whatmore and Reed, 1990; Cayley et al., 2000; Yao et al., 2002; Holland and Walsby, 2009; Deng et al., 2011; Mika et al., 2014). Cells adapt to changes in medium osmolality on the time scale of minutes to hours to maintain volume and crowding homeostasis (van den Berg et al., 2017). Bacterial cells exposed to hyperosmotic stress restore their volume by accumulating K^+^ ions and compensating anions and compatible solutes (Wood, 1999, 2011; Bremer and Krämer, 2019).

With the development of novel microscopy techniques and improvement of fluorescent probes, various methods are available to determine the lateral diffusion of molecules in the cell and to get insight into the dynamic structure of the cytoplasm (distinct phases, confined spaces, transient interactions between molecules) (Selvin, 2000; Sprague et al., 2004; Wawrezinieck et al., 2005; Hess et al., 2006; Fu et al., 2010). The method of fluorescent recovery after photo-bleaching (FRAP) is most often used to probe the diffusion of molecules; variations on FRAP approaches such as pulsed-FRAP, and whole-cell-FRAP can be advantageous for specific applications (Elowitz et al., 1999; Konopka et al., 2006; Mullineaux et al., 2006; van den Bogaart et al., 2007). In this paper, we employ conventional FRAP to determine the mobility of GFP (anionic and cationic derivatives) in *L. monocytogenes*, a Gram-positive bacterial pathogen.

*L. monocytogenes* is the causative agent for the foodborne infection listeriosis, which is a particular threat for pregnant, elderly, or immunocompromised individuals (Cossart and Toledo-Arana, 2008; Radoshevich and Cossart, 2018). *L. monocytogenes* is well-known for its resilience to osmotic challenges. For instance, the upper limit for growth under osmotic stress in the brain heart infusion (BHI) and chemically defined medium (CDM) are 2 and 1 M NaCl, respectively, which in CDM could be expanded to 1.5 M NaCl when the medium was supplied with the compatible solute glycine betaine (Amezaga et al., 1995). In *L. monocytogenes*, SigB plays essential roles in the general stress response, resistance to acid, oxidative and osmotic stress, growth at low temperatures, and the response to carbon starvation (Becker et al., 1998, 2000; Wiedmann et al., 1998; Ferreira et al., 2001; Fraser et al., 2003; Wemekamp-Kamphuis et al., 2004); and SigB also contributes to the virulence of the organism (Kazmierczak et al., 2003; Sue et al., 2004; Kim et al., 2005). While many studies have focused on the ability of this pathogen to survive different harsh environmental conditions (McClure et al., 1989; Walker et al., 1990; O’Driscoll et al., 1996; Chaturongakul et al., 2008; Chan and Wiedmann, 2009), little is known of the dynamic structure of the cytoplasm of *L. monocytogenes* under these conditions. The dynamics inside a bacterium can be assessed by the diffusion of proteins in its cytoplasm. Therefore, we determined the translational diffusion coefficients of GFP in *L. monocytogenes* as a function of temperature and osmotic stress, using wild type and stress-sensitive σ*^*B*^* null (Δ*sigB*) strains. We also measured the volume and turgor pressure as these parameters are connected to macromolecular crowding and diffusion in the cell.

## Materials and Methods

### Strains and Plasmids

We used *L. monocytogenes* EGD-e, wild-type and Δ*sigB* strain. The *L. monocytogenes* EGD-e Δ*sigB* mutant strain was constructed by allelic replacement of the wild-type gene by homologous recombination, using the integrative shuttle vector pMAD (Arnaud et al., 2004). The deletion of 561 bases of the *sigB* gene was confirmed by whole-genome sequencing and the Δ*sigB* strain does not possess any additional mutations in the chromosome compared to the isogenic parental strain (Marinho et al., 2019; Guerreiro et al., 2020). The green fluorescent protein (GFP) was expressed from the constitutive promoter P*_*dlt*_* using vector pNF8, which was provided by Tine Rask Licht at the Technical University of Denmark (Andersen et al., 2006). In the original paper, the vector bears the *gfp-mut1* variant of GFP (Fortineau et al., 2000). However, according to the sequencing data (Supplementary Materials), we obtained the *gfp-mut3b* variant (Cormack et al., 1996), which has a net surface charge of −8 (at pH 7.5). We also constructed a vector expressing a GFP derivative with an overall surface charge of +25, namely pNFpos25GFP. Here, the *gfp-mut3b* gene of pNF8 was replaced by the +25*gfp* gene present in pBAD +25GFP (Schavemaker et al., 2017), using the USER^®^ fusion method (Geu-Flores et al., 2007); the GFP +25 variant has an N-terminal his-tag. Competent *L. monocytogenes* cells were prepared and cells were transformed using the protocol previously described by Monk et al. (2008).

### Preparation of Cells for FRAP Measurements

All cultures have been handled at the biosafety level II (BSLII). In brief, glycerol stocks of *L. monocytogenes* stored at −80°C freezer were inoculated in 3 mL Brain Heart Infusion (BHI) medium in 10 mL tubes and incubated overnight at 30°C with 200 rpm shaking. Unless indicated otherwise, the cultures for the FRAP measurements were supplied with erythromycin (15 μg/mL) and nalidixic acid (100 μg/mL) (Fortineau et al., 2000). On day 2, the culture in the stationary phase was diluted 1:250 into a fresh pre-warmed medium (BHI or chemically defined medium, CDM) and incubated under the same conditions. The CDM is based on the recipe of Amezaga et al. (1995), and the medium was supplemented with 0.4% w/v glucose. On day 3, the culture was diluted again in the fresh and pre-warmed medium to obtain OD_600_ 0.15–0.2. The new culture was then incubated at 30°C with 200 rpm shaking until the OD_600_ reached 0.3–0.8 and thereafter used for FRAP measurements. All cultures were grown in 10 mL culturing tubes, and each FRAP experiment was repeated at least three times (independent biological replicates).

To determine the effect of medium osmolality, cells from an overnight culture in CDM were transferred to CDM supplemented with varying concentrations of NaCl. Osmolality values were measured with an Osmomat 030 cryoscopic osmometer (Gonotec, Berlin, and Germany). The osmolality of freshly prepared CDM medium is 0.23 Osm and was increased to maximally 2.37 Osm by the addition of NaCl. The cultures grown at higher osmolality required a longer time to reach the mid-exponential phase, and therefore, we adjusted the time of harvesting accordingly. For the osmotic shock experiments, cells from an overnight culture in CDM were diluted in CDM (0.23 Osm) and grown to the mid-exponential phase. Next, the cells were osmotically shocked in CDM supplied with NaCl and immediately prepared for microscopy experiments, which were completed within 30 to 50 min after the osmotic upshift.

To determine the effect of temperature, the cells were grown on day 1 and 2 at 30°C, and on day 3 at OD_600_ of 0.3–0.4 the cultures were brought to the new temperature and allowed to continue growing until OD_600_ 0.6–0.8. At 7°C and 42°C, the cultures were shaken at 60–80 rpm (Angelidis and Smith, 2003). In case of a temperature up- or downshift (temperature shock), the cells were grown at 30°C to an OD_600_ of 0.6–0.8, after which they were transferred to an icebox (4–7°C) or heat block (37–48°C). The cells were analyzed under the microscope within 30 to 50 min after the temperature shock. The temperature of the microscopy stage and the objective were the same as the temperature of the last step in the cultivation or temperature shift. We used a climate chamber at the microscope for temperatures higher than room temperature and a custom-made silicon-tubing system for cooling below room temperature.

### Glass Slide Preparation for Cell Immobilization

Cell immobilization on a glass slide is essential for FRAP measurements. We used (3-aminopropyl)triethoxysilane (APTES) to treat the glass slides. Glass slides were first cleaned by sonication in 5 M KOH for 1 h and rinsed thoroughly with Milli-Q. The slides were dried using an air gun, and the surface was activated with oxygen plasma (65 W) for 1 min. Then, the slides were deposited in 1% APTES in acetone and incubated for 20 min at room temperature. The glass slides were cleaned thoroughly by Milli-Q water and finally dried by pressurized nitrogen. The treated glass slides have primary amine groups on the surface that allow the binding of cells. After placing the cells on the glass slide, a coverslip was placed on top, and the system was sealed for biosafety purposes (BSLII) using a coverslip Sealant (Biotium). The slides were transferred to the microscopy facility and measurements were done immediately on the stand-by microscope.

### FRAP Acquisition and Data Analysis

**FIGURE 1:**
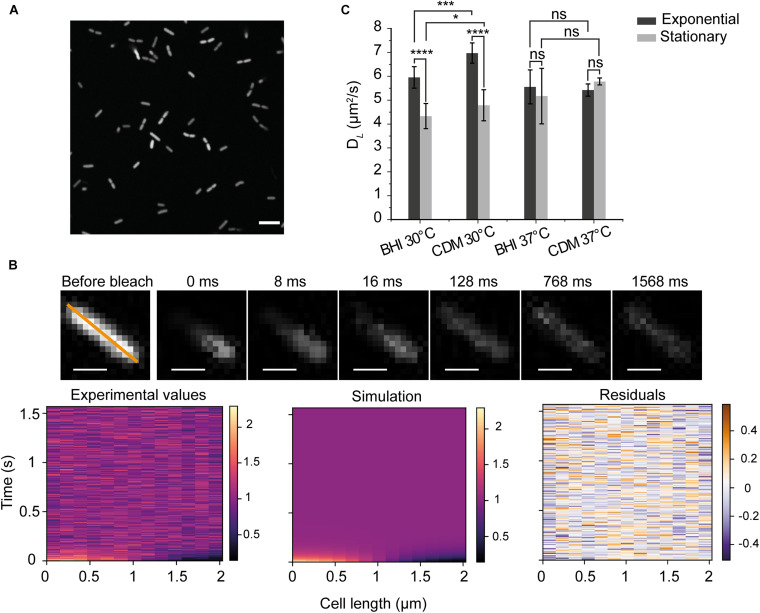
Principle of diffusion measurements in *Listeria monocytogenes* **(A)** An image of *L. monocytogenes* EGD-e/pNF8 expressing –8 GFP (scale bar 5 μm). **(B)** Data from FRAP acquisition. We show an example of the recovery of fluorescence for the diffusion constant of 6.8 ± 0.3 μm^2^/s. The orange line marks the analyzed region; each acquisition has 200 cycles, and the average intensity before bleaching (the first three cycles) was used for the normalization of the images. Time zero was recorded immediately after the photobleaching (scale bar 1 μm). Three graphs below indicate the fluorescent intensity along the orange line of the labeled cell in time, for the experimental data (left), the one-dimensional heat-equation simulation (middle), and the residuals (right). **(C)** *D*_*L*_ of –8 GFP for cells grown in BHI and CDM and analyzed in the exponential and stationary phase. Standard deviation (SD) is based on the means of three replicate datasets. There were more than 30 cells analyzed in each replicate dataset and more than 100 cells for each conditions. One-way ANOVA test (*p* < 0.05) and Fisher LSD *post hoc* tests were applied for all measured cells. *significance at *p* < 0.05; ***at *p* < 0.001; ****at *p* < 0.0001 and ns, not significant.

We used a Zeiss LSM710 confocal laser-scanning microscope (Zeiss, Oberkochen, Germany) with a C-apochromat 40x water immersion objective with NA of 1.2 for the acquisition of FRAP data. The measurements are similar to those described by Elowitz et al. (1999) with modifications described previously (Mika et al., 2014; Schavemaker et al., 2017). In brief, both the photobleaching and imaging were conducted at 488 nm, a high-intensity laser pulse was used for photobleaching, and a low-intensity laser for imaging of the cells. The fluorescent emission was collected from 493 to 700 nm. An ideal field of view has several discrete cells expressing GFP (Figure 1A). We took high-resolution (512 × 512 pixels) zoom-ins of cells to assure that (i) they flatly adhered to the glass surface; (ii) the fluorescent signals are homogeneously distributed; and (iii) the cells are not dividing. A square box was drawn over half of a cell at one of the poles designated as the bleaching area. Next, the resolution was decreased to 16 × 16 pixels before starting the acquisition to enable fast scanning and monitor fast fluorescent recovery. For FRAP measurements, the images were recorded for 200 cycles (total time of about 1.6 seconds with time intervals of 8 ms).

The diffusion coefficients were calculated from the FRAP acquisition, as reported before (Mika et al., 2014). The home-written software was converted to Python language, executed by Fiji (ImageJ) and Python 3.7.1. The analysis was done in batch, with many cells done in parallel. Briefly, a line is automatically drawn along the long axis of the cell. The fluorescent profiles along the line are extracted for each frame. The fluorescence intensity before photobleaching was used to normalize the measured distributions (Figure 1B). To obtain the lateral diffusion coefficient (*D*_*L*_), a script was used to simulate the normalized fluorescence intensity distributions along the drawn line during the recovery process. The residuals displaying the differences between the actual data and simulated data are reported in Figure 1B. We employ the one-dimensional (1D) continuous diffusion equation for the simulation, which is given by:

$$\frac{\partial I\,\left(x,t\right)}{\partial t}\,=D_{L}\,\frac{\partial^{2}I\,\left(x,t\right)}{\partial x^{2}}$$

With boundary condition:

$$\frac{\partial I\,\left(x,t\right)}{\partial t}\,=0$$

Where *I* is the fluorescence intensity, and *D*_*L*_ is the diffusion coefficient. The 1D diffusion simulation in Python is based on the heat diffusion equation of the Crank-Nicolson scheme (Crank and Nicolson, 1947).

### Cell Size Measurements

We used the wide-field microscope Zeiss Axio Observer (Zeiss, Oberkochen, Germany) in phase contrast (Ph3) mode, using the Plan-Apochromat 100x, oil immersion with NA of 1.4 objective to capture images of the cells. Cell immobilization on glass slides was done as described above. The length and width of cells were extracted by using the plugin MicrobeJ from ImageJ (Ducret et al., 2016). The volume (*V*) of the cell is given by:

$$V=\pi \,\frac{w^{2}}{4}\,\left(l-\frac{w}{3}\right)$$

where *w* is the width, and *l* is the length of the cell. Here, one assumes that the geometry of *L. monocytogenes* is described by a hemispherical cylinder.

### Computational Analysis of Proteome

The protein sequences from *L. monocytogenes* EGD-e (proteome ID: UP000000817) were retrieved from the UniProt server (Glaser et al., 2001). The pre-computed isoelectric point (pI) of each protein was calculated from the amino acid sequence, using the Isoelectric Point Calculator (IPC), was obtained from the Proteome Isoelectric Point Database (Kozlowski, 2016, 2017). We adapted the original IPC program to calculate the protein net charge based on the IPC_protein pKa dataset (Schavemaker et al., 2017). A pH value of 7.5 was chosen for the protein net charge calculation (Fang et al., 2004). We utilized the modified program to calculate the net charge of the GFP variants used in this work and to characterize the overall proteome of *L. monocytogenes*.

Histograms of the distribution of the isoelectric points (pI) and net charge of all proteins encoded by the whole genome and cytoplasmic subcellular localization of *L. monocytogenes* EGD-e were drawn. The cytoplasmic proteins are separated from the whole proteome based on a comprehensive secretomics-based subcellular localization database, which is available for *L. monocytogenes* EGD-e (Renier et al., 2012), together with the predictions from location prediction tools for bacterial proteins such as LocateP (Zhou et al., 2008) and SurfG+ (Barinov et al., 2009).

## Results

### Diffusion of GFP in the Cytoplasm of *Listeria monocytogenes*

We first measured the diffusion coefficient of GFP in the cytoplasm of *L. monocytogenes* EGD-e grown aerobically either in the complex brain heart infusion (BHI) or the chemically defined medium with glucose as the carbon source (CDM). The maximal growth rates (μ*_*MAX*_*) of *L. monocytogenes* EGD-e at 30°C are 0.82 ± 0.04 and 0.33 ± 0.03 h^–1^ for BHI and CDM, respectively; while the Δ*sigB* strain grows faster with μ*_*MAX*_* values of 0.91 ± 0.04 and 0.37 ± 0.04 h^–1^ for BHI and CDM, respectively. The net charge of GFP expressed from the pNF8 vector is −8 at pH 7.5. Figure 1A shows that GFP is homogeneously expressed with no indications of aggregation. A typical FRAP experiment and accompanying data analysis is illustrated in Figure 1B. In general, we found the diffusion coefficient of GFP in the cytoplasm of *L. monocytogenes* varied from approximately 4.5–7 μm^2^/s. Remarkably, we find statistically significant differences in the mobility at 30°C for cells in the exponential and stationary phase in both BHI and CDM (*p* < 0.0001) (Figure 1C). There are no significant differences in *D*_*L*_ of GFP for cells grown at 37°C (Detailed statistics in Supplementary Table 1). Generally, the *D*_*L*_ in cells grown in CDM are higher than those in BHI-grown cells at 30°C, which may relate to differences in the osmolality of BHI (0.44 Osm) and CDM (0.23 Osm).

### Diffusion of Anionic Versus Cationic GFP

**FIGURE 2:**
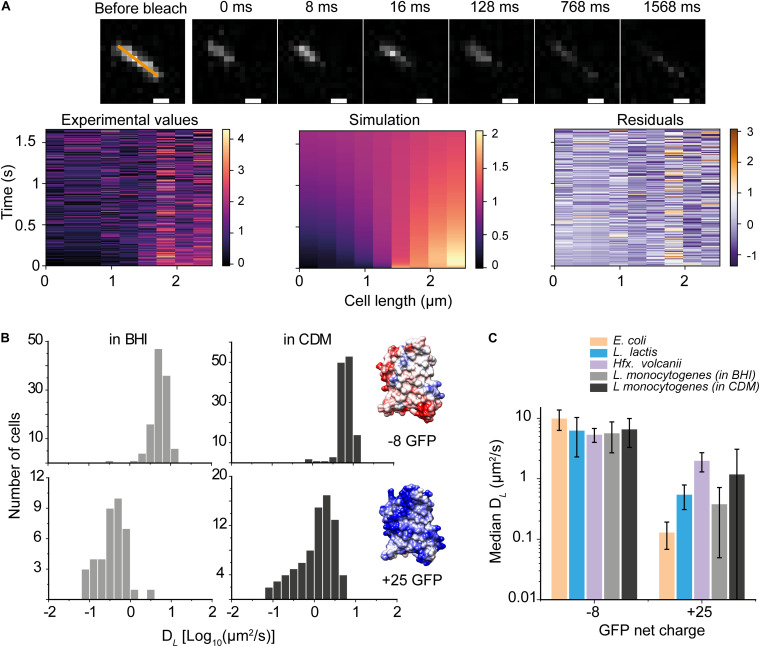
Diffusion of anionic and cationic GFP in *Listeria monocytogenes*. **(A)** (top) Recovery of +25 GFP fluorescence, corresponding to *D*_*L*_ of 0.81 ± 0.042 μm^2^/s. (bottom) The fluorescent intensity along the orange line is shown as a function of time for the experimental data (left) and the one-dimensional heat-equation simulation (middle), and the residuals of the data (right). **(B)** Histograms of diffusion coefficients of –8 GFP (top) and +25 GFP (bottom) of cells grown in BHI and CDM as well as structural models the surface-modified GFP variants; the colors display the surface charge. The models are based on the structure of super-folder GFP (PDBID: 2B3P), and the images were created using UCSF Chimera (Pettersen et al., 2004). Poisson-Boltzmann electrostatics calculations and evaluations were done by PDB2PQR and APBS packages (Baker et al., 2001; Dolinsky et al., 2004). **(C)** The diffusion coefficient of –8 and +25 GFP in *L. monocytogenes*, and –7 and +25 GFP in *E. coli, L. lactis*, and *Hfx. volcanii;* the latter have been taken from Schavemaker et al. (2017). The bars indicate medians and the error bars show the interquartile range.

Next, we used a GFP variant with a net charge of +25 at pH 7.5 and determined its mobility in cells grown in BHI and CDM (Figures 2A,B). We find that D*_*L*_* of +25 GFP is one to two orders of magnitude slower than that of −8 GFP; we also find a broader distribution of diffusion coefficients for +25 GFP (Figure 2B), suggesting that +25 GFP is likely interacting transiently with macromolecules in the cytoplasm. Figure 2C benchmarks the (*D*_*L*_) −8 GFP and +25 GFP in *L. monocytogenes* against similar measurements in *E. coli, L. lactis*, and *Hfx. volcanii* (taken from Schavemaker et al. (2017); the median values are plotted, and the error bars show the interquartile ranges (IQR). We see that the (*D*_*L*_) −8 GFP is comparable in the four microorganisms, whereas the lateral diffusion coefficient of +25 GFP in *L. monocytogenes*, particularly for cells grown in BHI, is faster than in *E. coli* but slower than in *Hfx. volcanii.* We consistently observed faster diffusion of proteins in *L. monocytogenes* grown in CDM than in BHI (Figure 2C).

**FIGURE 3:**
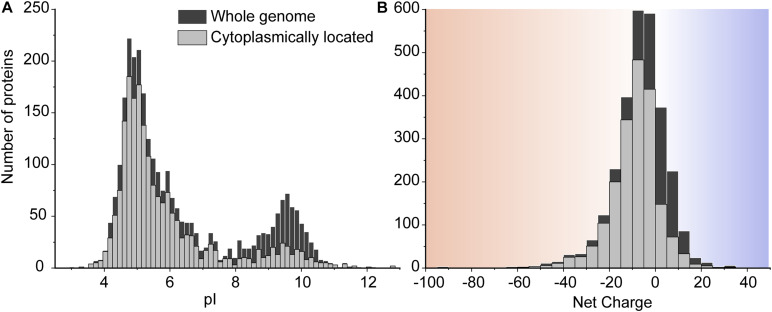
The pI **(A)** and net charge **(B)** distributions for proteins of *L. monocytogenes* EGD-e. The histograms show the number of genes that encode proteins for the whole genome scale and the proteins located in the cytoplasm. We used a pH of 7.5 to calculate the net charge. The cytoplasmic proteins are extracted from the whole proteome based on the secretomics-based subcellular localization database (Renier et al., 2012). To reveal more details in the histogram of the net charge, we removed four super-charged proteins which are outliers in the protein net charge distribution. These are three super-negatively charged cell-wall proteins including a putative peptidoglycan bound protein (with UniProt entry Q8Y697, *Z* = –241), a peptidoglycan anchored protein (Q8Y479, *Z* = –140), and Internalin I protein (Q8YA32, *Z* = –112); and one super-positively charged protein predicted to be on the cell membrane, a putative tape-measure protein of bacteriophage A118 (Q8Y4Z2, *Z* = 82).

The consequences of slower diffusion of cationic GFP in the cytoplasm were evaluated in the light of the proteome composition of *L. monocytogenes* EGD-e, similar to that described previously for other microorganisms (Schavemaker et al., 2017). We computed the pI and net charge values of all proteins from *L. monocytogenes* EGD-e at pH 7.5 (Figure 3). There are 2,844 genome-encoded proteins, of which 1,941 (∼68%) are cytoplasmic as predicted by secretomics (Renier et al., 2012). We see that most proteins have pI values between 4 and 7 and thus a net negative charge at typical internal pH values of *L. monocytogenes* (Fang et al., 2004). There are 2,111 negatively-charged proteins (74%) and 733 positively-charged proteins (26%) in the whole genome. In the cytoplasm, the percentage of negatively-charged is 86%. Of the 270 positively charged cytoplasmic proteins, there are 50 with a charge larger than +10 and potentially having a surface that would allow them to bind to anionic surfaces. Indeed, 27 out of 50 are predicted to be ribosomal proteins, nine are DNA-binding, six are RNA-binding, three are known enzymes, and five are uncharacterized proteins. Thus, the vast majority of highly positively charged proteins are part of nucleoprotein assemblies, and their cationic surfaces are required for complex formation and may not cause unwanted interactions with other macromolecules. The anionic cytoplasmic proteome of *L. monocytogenes* warrants relatively fast diffusion in the crowded environment of the cell. More details of the proteome analysis can be found in the Supplementary Information.

### Effect of Osmotic Stress on Protein Diffusion, Cell Volume, and Turgor Pressure

**FIGURE 4:**
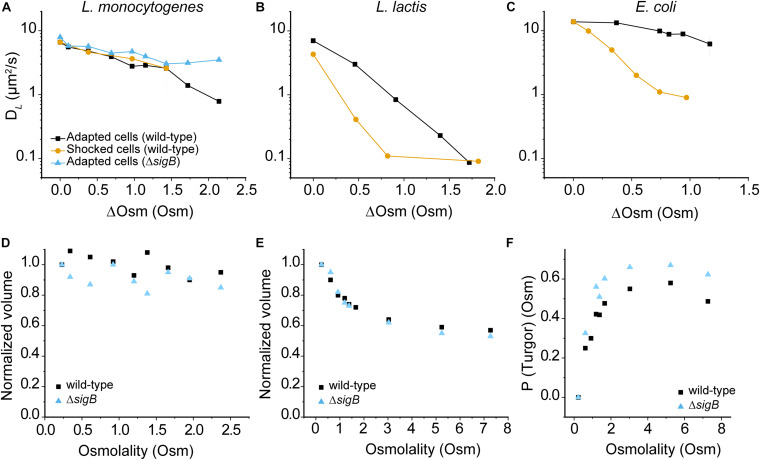
Effect of hypertonicity on the diffusion of GFP, cell volume and turgor pressure. **(A)** *D*_*L*_ of GFP in osmotically-adapted and shocked cells of *L. monocytogenes* EGE-e and EGD-e Δ*sigB*. Panels **(B)** and **(C)** show similar data for *L. lactis* and *E. coli*, taken from Konopka et al., 2009 and Mika et al., 2014. Median values of D*_*L*_* are used for *L. monocytogenes* and *L. lactis;* and mean values for *E. coli.* Details of interquartile ranges and standard deviations are presented in the Supplementary Table 2. In all cases cells were grown in chemically defined media with initial medium osmolalities of 0.23, 0.53, and 0.28 Osm for *L. monocytogenes, L. lactis*, and *E. coli*, respectively; ΔOsm reflects the addition of NaCl to these media. **(D)** and **(E)** panels show the normalized volume of *L. monocytogenes* EGD-e and EGD-e Δ*sigB* of osmotically-adapted **(D)** and shocked cells **(E)**; a minimum of 100 (up to 800) cells were analyzed for each condition. A value of 1 corresponds to a volume of 0.61 μm^3^ and 0.76 μm^3^ for *L. monocytogenes* EGD-e wild-type and Δ*sigB* cells, respectively. **(F)** Turgor pressure plots of *L. monocytogenes* EGD-e wild-type and Δ*sigB*.

We determined the effect of hypertonic conditions on the diffusion of −8 GFP in osmotically-adapted and shocked cells. We grew cells in CDM medium and use CDM supplied with NaCl for osmotic upshifts. Remarkably, we find no difference in diffusion between adapted and shocked cells contrary to what is seen in *L. lactis* and *E. coli* (Figures 4A–C), suggesting that *L. monocytogenes* is rather insusceptible to osmotic upshift ranges used in this study. Data for *L. lactis* and *E. coli* are taken from Konopka et al., 2009 and Mika et al., 2014, and cells were grown in chemically defined media with initial medium osmolalities of 0.23, 0.53, and 0.28 Osm for *L. monocytogenes, L. lactis*, and *E. coli*, respectively. Taken together, in osmotic upshift measurements, *D*_*L*_ values for GFP in the cytoplasm remain comparable suggesting a unique tolerance to osmotic stress of *L. monocytogenes* EGD-e. The data for the Δ*sigB* strain are similar to wild-type *L. monocytogenes*, except at the highest osmolalities where the Δ*sigB* strain seems even less affected in protein mobility. This suggests a difference in the intracellular environment between two strains due to the control of SigB to its regulon when subjected to significantly high salt media. We show the distributions of *D*_*L*_ values for GFP in the cytoplasm of *L. monocytogenes* EGD-e and Δ*sigB* cells grown in chemically defined media (CDM) at different osmolality in the histograms in Supplementary Figure 1, 2.

We wondered why an osmotic upshift affects −8 GFP diffusion in *L. monocytogenes* much less than in *L. lactis* or *E. coli* (−7 GFP). Figure 4D shows that the volume of adapted *L. monocytogenes* cells drops at most 10%, and the instantaneous drop in volume upon osmotic upshift Figure 4E is also much less than in *L. lactis* or *E. coli*. Also, we did not observe any cellular invaginations typical of plasmolyzing *E. coli* cells (Koch, 1998; Konopka et al., 2006; Lewenza et al., 2006; Rojas et al., 2014). We noted that the volume of *L. monocytogenes* EGD-e Δ*sigB* (0.76 ± 0.26 μm^3^; *N* = 370) is significantly larger than that of wild-type cells (0.61 ± 0.21 μm^3^; *N* = 325) grown in CDM. We illustrate the changes in cell morphology that accompany the changes in cell volume upon osmotic upshift and subsequent adaptation phase in Supplementary Figure 4A; representative phase-contrast microscopy images are shown in Supplementary Figure 4B. We note that while a width remains constant following osmotic upshift, a significant decrease in length occurs and this affects the cell volume.

Next, we determined the turgor pressure (*P*_*turgor*_) of *L. monocytogenes* by applying the osmotic upshift method as described by Whatmore and Reed, 1990. The internal osmolality calculation uses the relationship: *P*_*turgor*_=π_*int*_−π_*ext*_, where π_*int*_ is the internal osmolality and π_*ext*_ is the external osmolality. Above a threshold level of the external osmolality, *P*_*turgor*_=0, and π_*int*_=π_*ext*_, and the cell should act as an ideal osmometer in accordance with the Boyle-van’t Hoff relationship (π*V*=*constant*). Thus, by calculating π_*int*_ at *P*_*turgor*_=0, this value can be used to determine π_*int*_at *P*_*turgor*_ > 0. The Boyle-van’t Hoff plot, i.e., cell volume versus reciprocal of the medium osmolality yields the non-osmotic volume (*V*^*NO*^), which is the intercept of the ordinate from the Boyle-van’t Hoff plot (Supplementary Figure 5). The *V*^*NO*^ values are 0.32 and 0.35 μm^3^ for the wild-type and Δ*sigB* cells, respectively. We can now calculate the osmotic volume (*V^*O*^)* at any π where *P*_*turgor*_=0 as *V*^*O*^ = *V*^*total*^ – *V*^*NO*^. By applying the Boyle-van’t Hoff relationship, we get *V^*O*^_*x*_*⋅π*_*x*_* = *V^*O*^_*m*_*⋅π*_*m*_*, where *V^*O*^_*x*_* and π*_*x*_* are the osmotic volume and internal osmolality at any point where *P*_*turgor*_=0; and *V^*O*^_*m*_* and π*_*m*_* are the osmotic volume and internal osmolality in the original medium (Whatmore and Reed, 1990). In Figure 4F, the turgor pressure at the plateau is the turgor pressure of the cells under growth conditions in the original medium (CDM). We estimate the turgor pressure of *L. monocytogenes* EGD-e and Δ*sigB* at 0.58 and 0.67 Osm (or ∼14.4 and ∼16.6 atm), respectively, which is in the range of that of *L. lactis* and *B. subtilis* (∼0.75 Osm) (Whatmore and Reed, 1990; Mika et al., 2014). Our results are in good agreement with previous measurements of the internal osmolality of *L. monocytogenes* (Patchett et al., 1992).

### Effect of Temperature on Protein Diffusion

*Listeria monocytogenes* is a foodborne pathogen capable of growing at temperatures as low as −0.4°C, whereas the maximal growth temperature is 45°C. Thus, we determined the mobility of −8 GFP of cells grown in complex BHI broth and CDM in temperatures ranging from 7 to 42°C.

**FIGURE 5:**
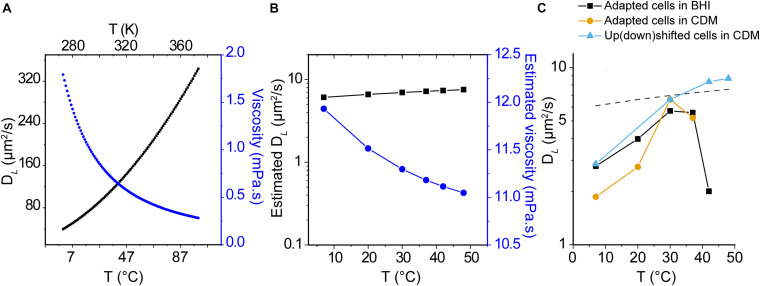
Temperature dependence of protein diffusion in *L. monocytogenes.* **(A)** Lateral diffusion coefficient of monomeric GFP with a Stokes radius R_*S*_ = 2.82 nm (Liarzi and Epel, 2005) in water at different temperatures (black line), as calculated using the Stokes-Einstein equation. The viscosity of water (μ) as a function of temperature (T) is shown as blue points. **(B)** Estimated viscosity of the cytoplasm and *D*_*L*_(GFP) as a function of temperature, using the experimental *D*_*L*_(GFP) at 30°C as starting value and assuming constant viscosity of the cytoplasm and constant interactions between the moving particle (GFP) and solvent, and assuming that the viscosity of water and cytoplasm have the same temperature dependence. **(C)** Median values for *D*_*L*_(GFP) for cells grown and analyzed at the indicated temperatures; cells were grown in BHI (black squares) and CDM (yellow circles). In another set of experiments the cells were grown in CDM at 30°C and diffusion of GFP was determined at the indicated temperature (turquoise triangles). The dashed line illustrates the estimated *D*_*L*_(GFP) of panel **(B)**. Details of interquartile ranges and statistical tests are presented in the Supplementary Table 3.

Lateral diffusion of globular proteins in an aqueous solution can be approximated by the Stokes-Einstein equation:

$$D_{L}\,=\frac{k_{B}\,T}{6\,\pi \,\mu \,R_{0}}$$

where *D*_*L*_ is the diffusion coefficient, *k*_*B*_ is Boltzmann’s constant, *T* is the temperature (K), μ is the solvent viscosity, and *R*_0_ is the radius of the protein. We used the Stokes-Einstein equation to estimate the diffusion coefficient of −8 GFP in aqueous media as a function of temperature and *D*_*L*_ (−8 GFP) in the cytoplasm at 30°C as the benchmark (Figure 5A). Furthermore, we used the Stokes-Einstein equation and *D*_*L*_(−8 GFP) at 30°C to get a gross estimate of the viscosity of the *L. monocytogenes* cytoplasm (Figure 5B), assuming constant viscosity of the cytoplasm and constant interactions between the moving particle (GFP) and solvent. None of these assumptions is correct, but the analysis allows a first comparison of the experimental data with the Stokes-Einstein model. We take the calculated viscosity of 11.3 mPa.s at 30°C as an approximation of the crowding in the cytoplasm. We then estimated the temperature dependence of the cytoplasmic viscosity and calculated *D*_*L*_(−8 GFP). As seen in Figure 5B, the diffusion coefficient increases moderately with temperature; *D*_*L*_(−8 GFP) increases from 6.1 to 7.5 μm^2^/s when the temperature increases from 7 to 48°C. Remarkably, our measurements of *D*_*L*_(−8 GFP) in temperature-adapted cells grown in BHI or CDM increases with the temperature reaching a maximum at around 30°C (Figure 5C). In CDM, the dataset is limited to 37°C because the cells didn’t grow at higher temperatures. The drop in mobility at higher temperatures is not seen when cells are grown at 30°C (*D*_*L*_ = 6.61 ± 3.30 μm^2^/s) and the diffusion is analyzed at 42°C (*D*_*L*_ = 8.34 ± 4.42 μm^2^/s) or 48°C (*D_L_* = 8.70 ± 6.53 μm^2^/s) (Median ± IQR, Figure 5C, triangle symbols).

Histograms of *D*_*L*_ in the cytoplasm of *L. monocytogenes* EGD-e grown at different conditions and analyzed at different temperatures are presented in Supplementary Figure 6. Supplementary Figure 7 shows *D*_*L*_ as a function of the acquisition time of three replicated datasets, which shows that the values are not affected over a measuring period of at least 1 h. In summary, the trend in protein mobility in the cytoplasm of *L. monocytogenes* does not follow the temperature dependence given by the Stokes-Einstein relationship. The structure or apparent viscosity of the cytoplasm appears to change above 30–40°C, an effect that is seen in adapted cells but not in cells exposed to a temperature upshift.

Finally, in this paper, we report the mean and median values of *D*_*L*_, which allows direct comparison with many other studies. The variation in the *D*_*L*_ values reflects the heterogeneity of cells within isogenic cultures, which increases when cells are stressed (Elowitz et al., 1999; Aertsen and Michiels, 2005; Lidstrom and Konopka, 2010). Elowitz et al. (1999) have estimated the cell-to-cell variation of *D*_*L*_ and find a 32% deviation from the mean in isogenic cultures. We come to a similar conclusion and find that differences between means and medians are mostly less than 0.5 μm^2^/s, or differ about 10% from each other (on the basis of about 3,700 measurements), that is in cells grown under non-stressed or low-level stress conditions.

## Discussion

In this paper, we probe the lateral diffusion of GFP in the cytoplasm of the Gram-positive pathogenic bacterium *L. monocytogenes*, and we benchmark our observations against *E. coli* and *L. lactis*. We choose *L. monocytogenes* because it is remarkably resistant to extreme stresses such as hypertonicity and capable of growth at temperatures well below 10°C, where organisms like *E. coli* and *L. lactis* do not grow. Besides, *L. monocytogenes* also survives the harshness of the host digestive tract and stresses imposed during the invasion, the translocation of the intestinal epithelial layer, and infection of other target organs in the human host. We analyzed the diffusion of −8 GFP in wild-type *L. monocytogenes* EGD-e and the stress-sensitive Sigma B (SigB or σ*^*B*^*) null strain (Δ*sigB)*. We grew and analyzed *L. monocytogenes* in complex BHI broth and chemical defined media (CDM), conditions typically used for physiological studies on *L. monocytogenes*.

We have analyzed the proteome of *L. monocytogenes* and find that the vast majority of cytoplasmic proteins are anionic and probably less hindered in their diffusion by electrostatic interactions than cationic proteins. We have previously shown for *E. coli, L. lactis*, and *Hfx. volcanii* that cationic proteins bind to ribosomes, which lowers the apparent diffusion coefficient by one to two orders of magnitude, depending on the ambient ionic strength of the cytoplasm (Schavemaker et al., 2017). We now observe that the diffusion +25 GFP is similarly reduced in *L. monocytogenes*, which we ascribe to binding of the protein to anionic surfaces as present on ribosomes. In *L. monocytogenes* 50 proteins have a net surface charge of +10 or higher, and more than 50% (26) of these are ribosomal proteins, which is even higher than in *E. coli* (18). The slowing of cationic GFP is less than in *E. coli* but more than in *Hfx. volcanii*, which most likely reflects the intermediate ionic strength.

When cells are exposed to high salt conditions, water will leave the cell, decreasing the volume. Cells then preferentially accumulate compatible solutes to increase the internal water concentration and thereby recover their volume (Ko et al., 1994; Amezaga et al., 1995; Verheul et al., 1997; Wood, 1999). When exposed to severe osmotic stress (>0.57 Osm), *E. coli* cells plasmolyze, which is observed as a lateral invagination of the cytoplasmic space (Konopka et al., 2006, 2009). These plasmolysis spaces are not seen in *L. monocytogenes*, but rather a decrease in cell size (mainly cell length) is observed. Further, the volume of *L. monocytogenes* decreases with increasing medium osmolality and remains devoid of plasmolysis spaces, even at 3.6 M of NaCl. Our observations indicate that *L. monocytogenes* can cope with severe hyperosmotic stress in CDM, even in the absence of added compatible solutes, conceivably due to its capacity to enlarge the intracellular pool of amino acids, as previously described by Amezaga et al. (1995).

The Δ*sigB* strain has a larger cytoplasmic volume and higher turgor pressure than the parental strain. This is a novel phenotype for the Δ*sigB* strain, but growth advantages under mild osmotic stress (0.5 M NaCl) (Abram et al., 2008), low-intensity blue light (O’Donoghue et al., 2016) and other mild stress conditions (Guerreiro et al., 2020) have previously been reported for Δ*sigB*. The SigB protein controls a large number of genes (>200) by binding to its promoters or neighboring regions (Gaballa et al., 2019). For example, in the stationary phase, SigB down-regulates genes involved in cell division (*fts* genes, division inhibitor *minD*), cell cycle control (*smc*), and cell wall biogenesis (*mreD, iap*, and *spl*) (Hain et al., 2008). The effects on cell division and cell cycle control may form the basis for the larger volume and higher turgor pressure of the Δ*sigB* strain.

The viscosity of aqueous solutions is well defined and can be experimentally determined (Viswanath et al., 2007), but the meaning of viscosity in the context of the crowded cytoplasm is less clear. Small molecules (osmolytes, compatible solutes) contribute to the micro-viscosity, but the diffusion of macromolecules will also be hindered by other macromolecules with which they may collide or transiently interact. Furthermore, the cytoplasm is not homogenous as certain macromolecules are excluded from the nucleoid (van den Bogaart et al., 2007). Following an osmotic upshift, the cytoplasm may even be compartmentalized. One can increase the macromolecular crowding, hence the “macro-viscosity,” by subjecting the cell to an osmotic upshift. We observe that protein mobility in *L. monocytogenes* is much less affected by an osmotic upshift than protein mobility in *E. coli* or *L. lactis*. This difference can be rationalized for *E. coli*, which has a much lower turgor pressure and already at a medium osmolality of 0.57 Osm, the cell plasmolyzes, and relative little solvent is left for diffusion (van den Bogaart et al., 2007). However, the turgor pressure of *L. monocytogenes* and *L. lactis* are similar and will decrease similarly when the external osmolality is increased. Yet, the impact of osmotic stress on the mobility of GFP in *L. monocytogenes* is much less, which is consistent with its higher stress tolerance compared to *L. lactis.* We have no simple mechanistic explanation for this difference since combinatorial effects at the cytoplasmic, membrane, and cell wall cannot be excluded and remain to be identified.

Many recent studies have shown an essential role of the second messenger c-di-AMP in the growth, cell wall biosynthesis, and osmoregulation of *L. monocytogenes* (Witte et al., 2013; Gibhardt et al., 2019). It is tempting to speculate that c-di-AMP plays a role in the ability of *L. monocytogenes* to resist osmotic stress as shown here by the relatively high mobility of proteins in the cytoplasm. We note that c-di-AMP is also present in *L. lactis* but not in *E. coli*, and thus there is not a simple correlation between resistance to osmotic stress and the regulation of the volume of these cells *via* the uptake and efflux of potassium ions and compatible solutes (Commichau et al., 2018; Gibhardt et al., 2020; Peterson et al., 2020; Sikkema et al., 2020). Besides, the physiological effect of c-di-AMP on the uptake of potassium in *L. monocytogenes* is less pronounced than in other *Firmicutes* (Gibhardt et al., 2019; Stülke and Krüger, 2020). Thus, the regulation by cyclic-di-AMP may not be the sole factor to explain the differences in osmotic stress resistance.

We are not aware of studies that report the temperature dependence of diffusion inside living cells even though the effect of temperature on the diffusion of lipids and proteins in membranes (Nenninger et al., 2014) and *in vitro* in media with synthetic crowders have been reported (Banks and Fradin, 2005). We find that *D*_*L*_ of cytoplasmic GFP increases about threefold when the temperature is increased from 7 to 30°C (Figure 5C), which is more than the number predicted by the Stokes-Einstein equation. At temperatures higher than 30°C, the *D*_*L*_ of cytoplasmic GFP decreases, but this effect is observed only in temperature-adapted cells and not in cells grown at 30°C and upshifted to a higher temperature (Figure 5C, blue triangles). The observed temperature effects above 30°C may arise from changes in the proteome composition of temperature-adapted cells, which may lead to a different cytoplasmic structure and composition, impacting protein diffusion. For example, the positive regulatory factor A (PrfA) in *L. monocytogenes* is a transcriptional activator that is thermally activated at 37°C and mediates the transcriptional reprogramming required to transition from a non-pathogenic to a pathogenic state (Johansson et al., 2002).

Not surprisingly, the Stokes-Einstein equation fails to predict the temperature-dependence of diffusion of proteins in heterogeneously crowded environments such as the bacterial cytoplasm. The current thinking is that weak, nonspecific interactions between the macromolecules of the cell slow their diffusion (Muramatsu and Minton, 1988; Zorrilla et al., 2007; Wang et al., 2010), which is dependent on the ambient proteome and metabolome. Deviations from Stokes-Einstein may also be caused by differences in binding equilibria, e.g., in many cases, the bound state is preferred at lower temperatures (Rothe et al., 2016). The macromolecular interactions in living cells change further when they are confronted with environmental insults. Our observations on the temperature dependence of protein diffusion in *L. monocytogenes* warrant further investigation in other cell types, not in the least because macromolecular viscosity or crowding is an important physicochemical factor in every living cell and temperature transients are common in many environments.

In summary, we have determined the lateral diffusion coefficient of GFP in the cytoplasm of *L. monocytogenes* under a range of physical and physiological conditions that influence the fitness and survival of the microorganism. Osmotic stress and a highly cationic surface of the target protein have significantly less impact on the diffusion in *L. monocytogenes* than it has in *E. coli* or *L. lactis.* Remarkably, the impact of osmotic stress is similar in shocked and adapted cells, and the temperature dependence of diffusion shows an optimum around the optimal growth temperature of *L. monocytogenes*. Further investigations, using additional mutants, may shed new light on the role of regulatory circuits and output signals on the structure of the cytoplasm in *L. monocytogenes*.

## Data Availability Statement

The datasets presented in this study can be found in online repositories. The names of the repository/repositories and accession number(s) can be found in the article/Supplementary Material.

## Author Contributions

BT, AI, and BP designed the study. BT conducted the experiments (with help from HP in studies on +25 GFP), analyzed the data, and wrote the first draft of the manuscript. BP, AI, CO, and TA supervised the work and edited the manuscript. All authors contributed to the article and approved the submitted version.

## Conflict of Interest

The authors declare that the research was conducted in the absence of any commercial or financial relationships that could be construed as a potential conflict of interest.
