# Supplementary material for: Measurement of Protein Mobility in Listeria monocytogenes Reveals a Unique Tolerance to Osmotic Stress and Temperature Dependence of Diffusion
Source: Front Microbiol. 2021 Feb 17;12:640149. doi: 10.3389/fmicb.2021.640149 (PMC7925416; doi:10.3389/fmicb.2021.640149)
Supplement: Supplementary file 1 [file Data_Sheet_1.pdf]

Supplementary Materials

Measurement of protein mobility in *Listeria monocytogenes* reveals a unique tolerance to osmotic stress and temperature dependence of diffusion

Buu Minh Tran[1], Haritha Prabha[1], Aditya Iyer[1], Conor O'Byrne[2], Tjakko Abee[3], Bert Poolman[1*]

[1]Department of Biochemistry, University of Groningen, Groningen, the Netherlands
[2]School of Natural Sciences, National University of Ireland, Galway, Ireland
[3]Laboratory of Food Microbiology, Wageningen University Research, Wageningen, the Netherlands

*** Correspondence:**
Email: b.poolman@rug.nl

1 Supplementary Data

Protein sequences

-8GFP
```
MSKGEELFTGVVPILVELDGDVNGHKFSVSGEGEGDATYGKLTLKFICTTGKLPVPWPTLVTTFGYGVQCFARY
PDHMKQHDFFKSAMPEGYVQERTIFFKDDGNYKTRAEVKFEGDTLVNRIELKGIDFKEDGNILGHKLEYNYNSH
NVYIMADKQKNGIKVNFKIRHNIEDGSVQLADHYQQNTPIGDGPVLLPDNHYLSTQSALSKDPNEKRDHMVLLE
FVTAAGITHGMDELYK
```

+25GFP
```
MGHHHHHHGGASKGERLFTGVVPILVELDGDVNGHKFSVRGKGKGDATRGKLTLKFICTTGKLPVPWPTLVTTL
TYGVQCFSRYPKHMKRHDFFKSAMPKGYVQERTISFKKDGTYKTRAEVKFEGRTLVNRIKLKGRDFKEKGNILG
HKLRYNFNSHNVYITADKRKNGIKANFKIRHNVKDGSVQLADHYQQNTPIGRGPVLLPRNHYLSTRSALSKDPK
EKRDHMVLLEFVTAAGITHGMDELYK
```

2 Supplementary Figures and Tables

2.1 Supplementary Figures

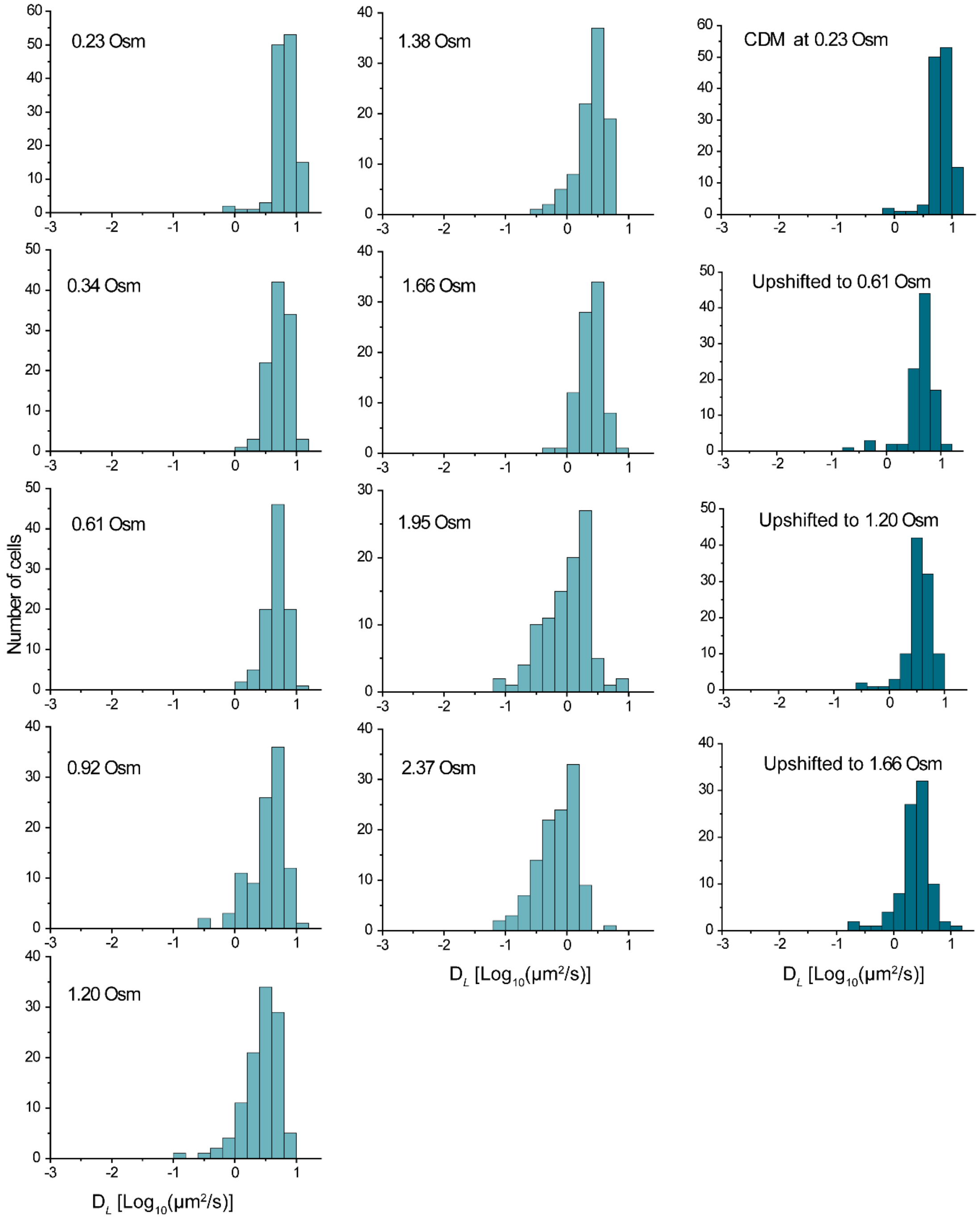

Supplementary Figure 1. Histograms of D_L(GFP) in the cytoplasm of *L. monocytogenes* EGD-e grown in chemically defined media (CDM) at different osmolality (adapted cells, final osmolality is indicated in the panels) or grown in CDM at 0.23 Osm and osmotically upshifted just prior to the measurements. The osmolality of CDM was increased by NaCl.

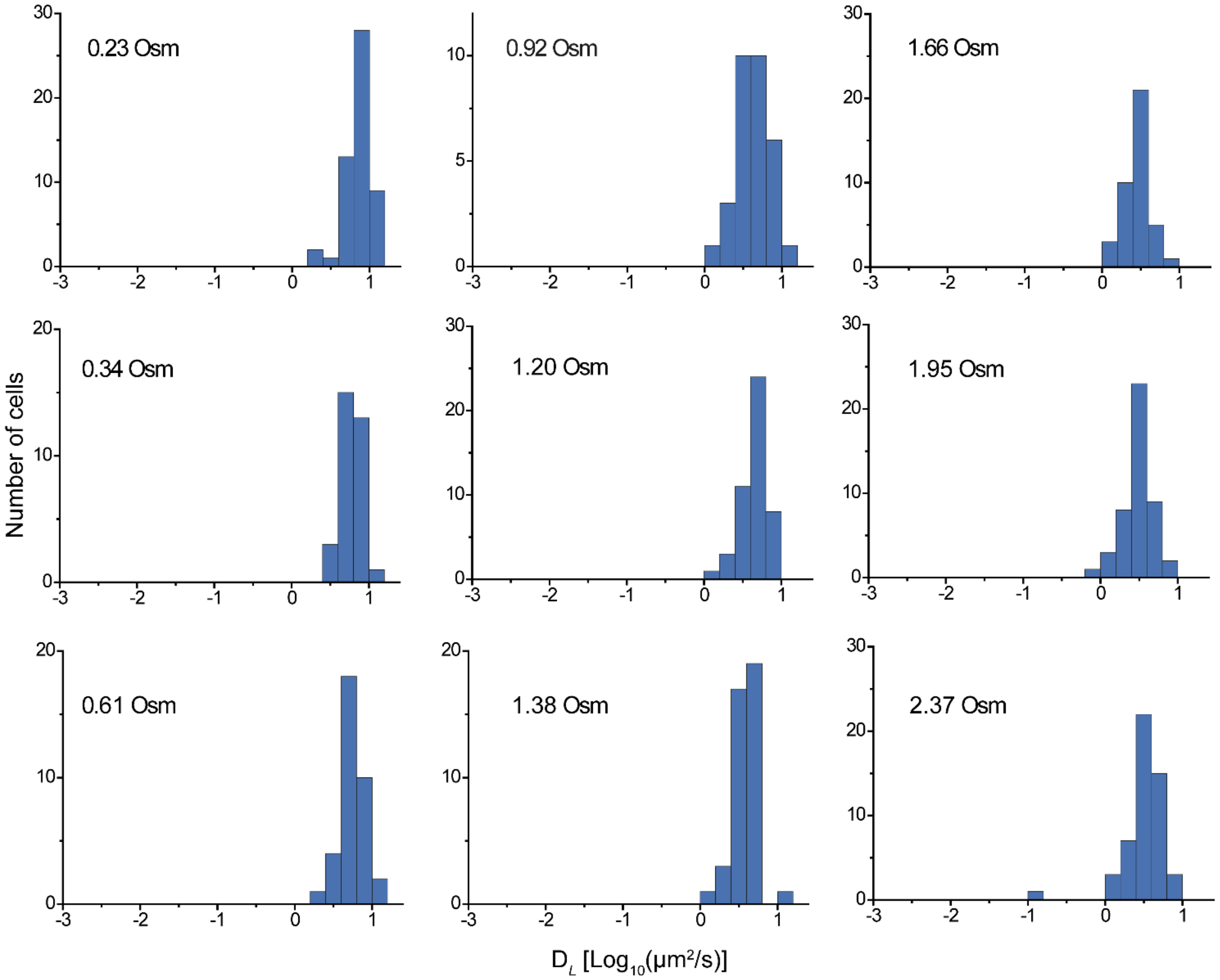

Supplementary Figure 2. Histograms of D_L(GFP) in the cytoplasm of *L. monocytogenes* EGD-e Δ*sigB* grown in CDM at different osmolality (adapted cells, final osmolality is indicated in the panels). The osmolality of CDM was increased by NaCl.

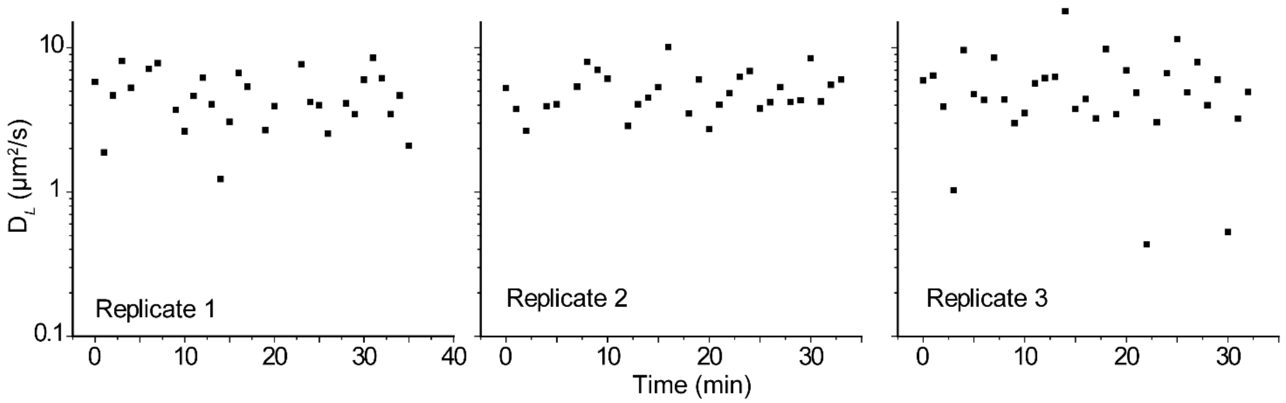

Supplementary Figure 3. Variation in D_L(GFP) plotted against the measuring time for three replicate samples of osmotically upshifted cells in CDM at 0.61 Osm. These plots display the D_L values obtained from individual cells against the measuring time.

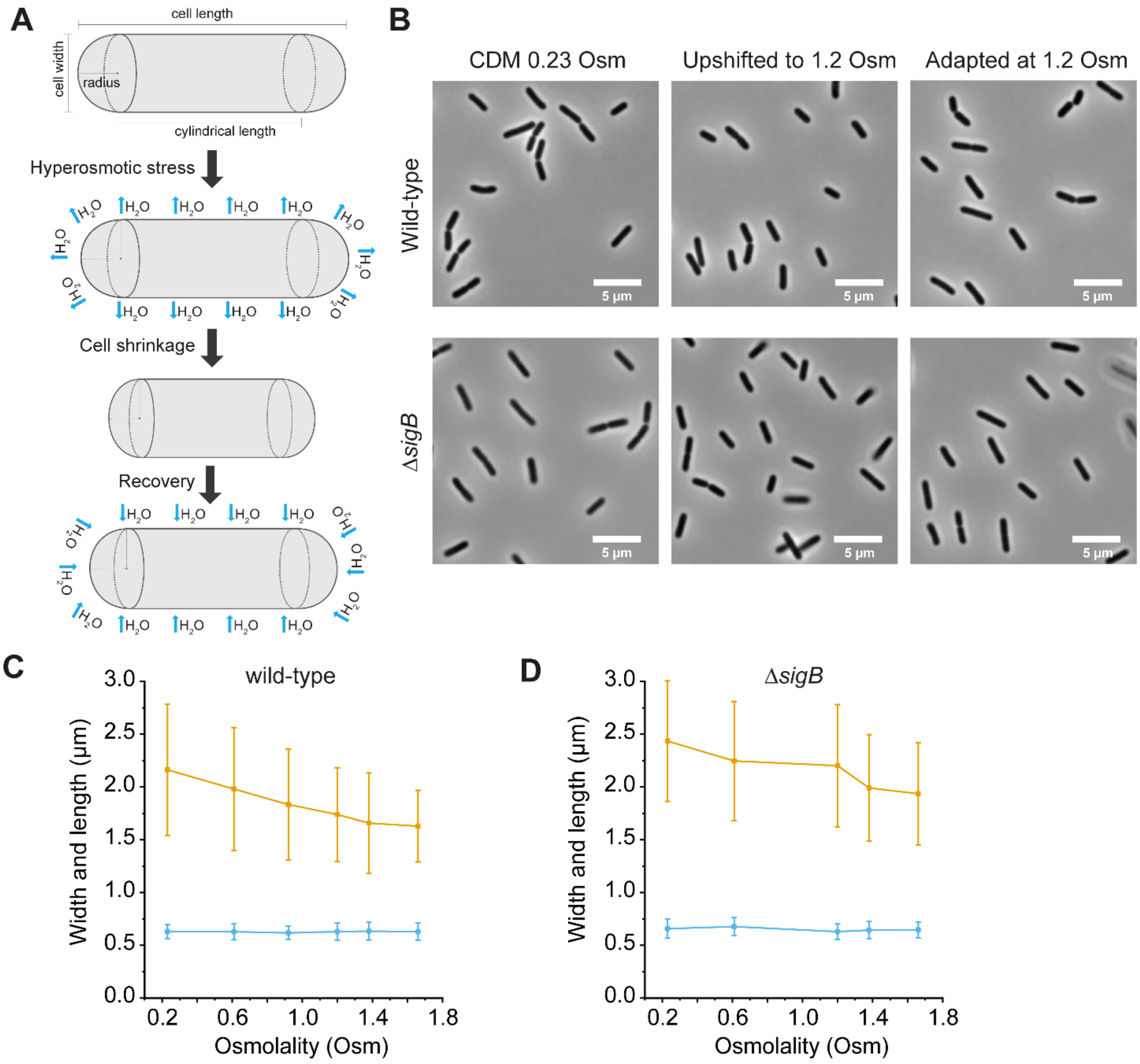

Supplementary Figure 4. *L. monocytogenes* cell morphology under hyperosmotic stress. (A) Scheme of hemispherical cylinder mimicking a rod-shaped cell and volume changes when the cell is subjected to hyperosmotic stress. (B) Typical phase-contrast images of *L. monocytogenes* cells at varying conditions of osmotic stress. Changes of the width (blue line) and length (yellow line) as a function of the osmotic upshift of wild-type (C) and Δ*sigB* (D) cells. While the width remains largely unchanged, the length of cells decreases when cells are osmotically upshifted.

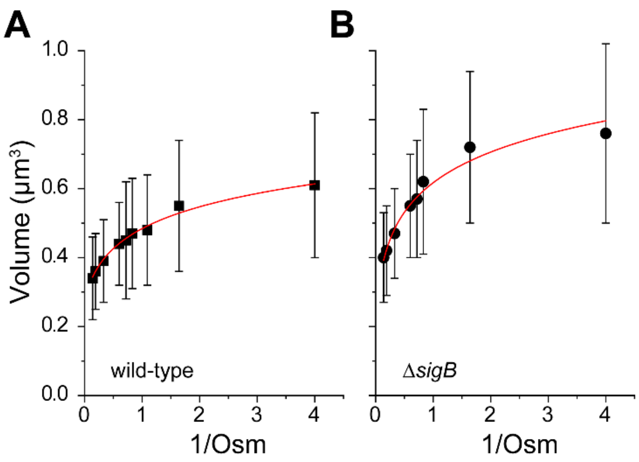

Supplementary Figure 5. Boyle-van't Hoff plots of *L. monocytogenes* EGD-e wild-type (A) and Δ*sigB* (B). The cell volume is plotted as function of the reciprocal of the medium osmolality. The intercept with the y-axis yields the non-osmotic volume (V^{NO}) of the cell, which is used to compute the turgor pressure of the cell as described in the main text.

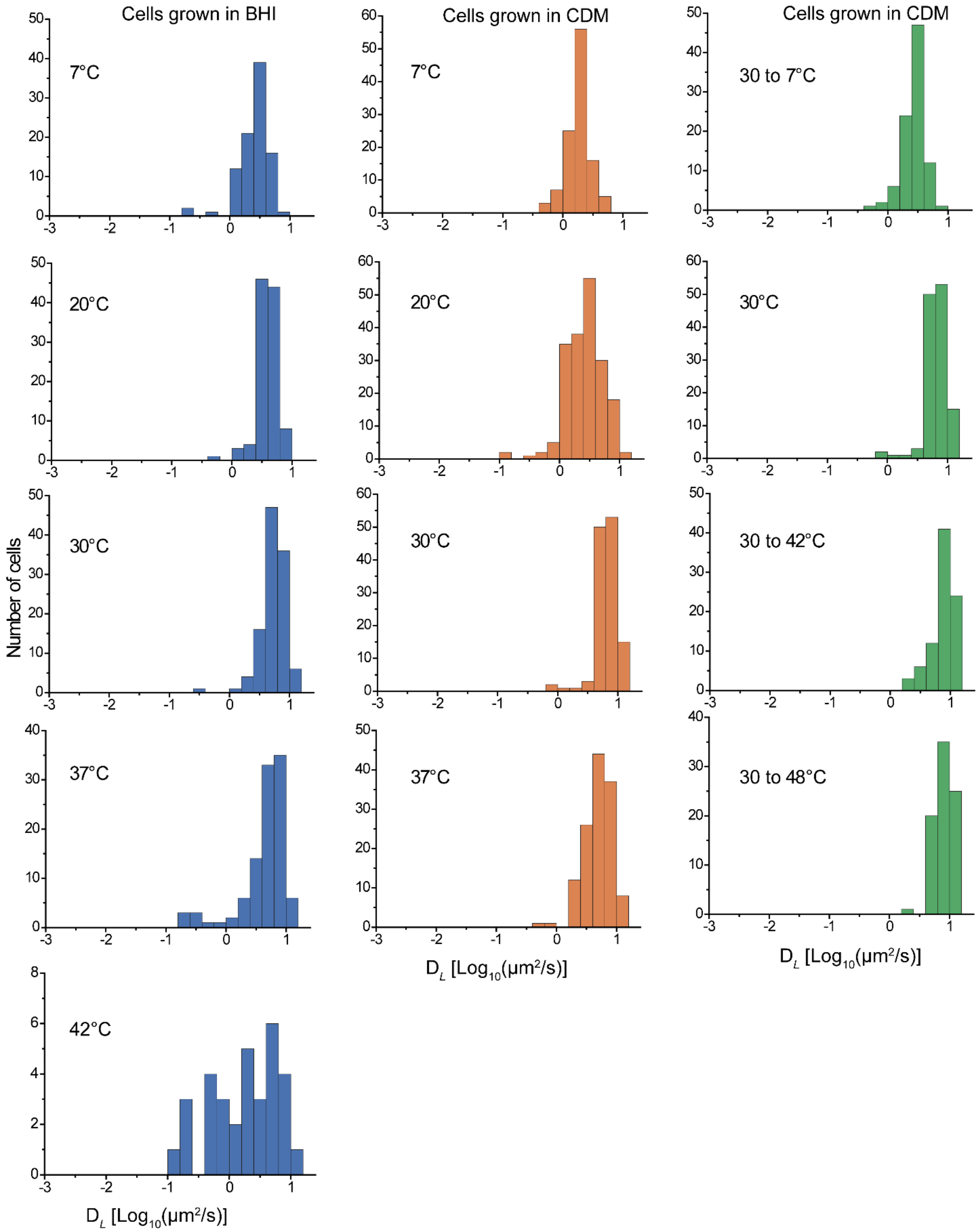

Supplementary Figure 6. Histograms of D_L(GFP*)* in the cytoplasm of *L. monocytogenes* EGD-e cells grown in BHI and CDM at different temperatures and analyzed at the indicated temperatures (left and middle panels) or grown at 30°C and shifted to a different temperature for analysis of the diffusion (right panels).

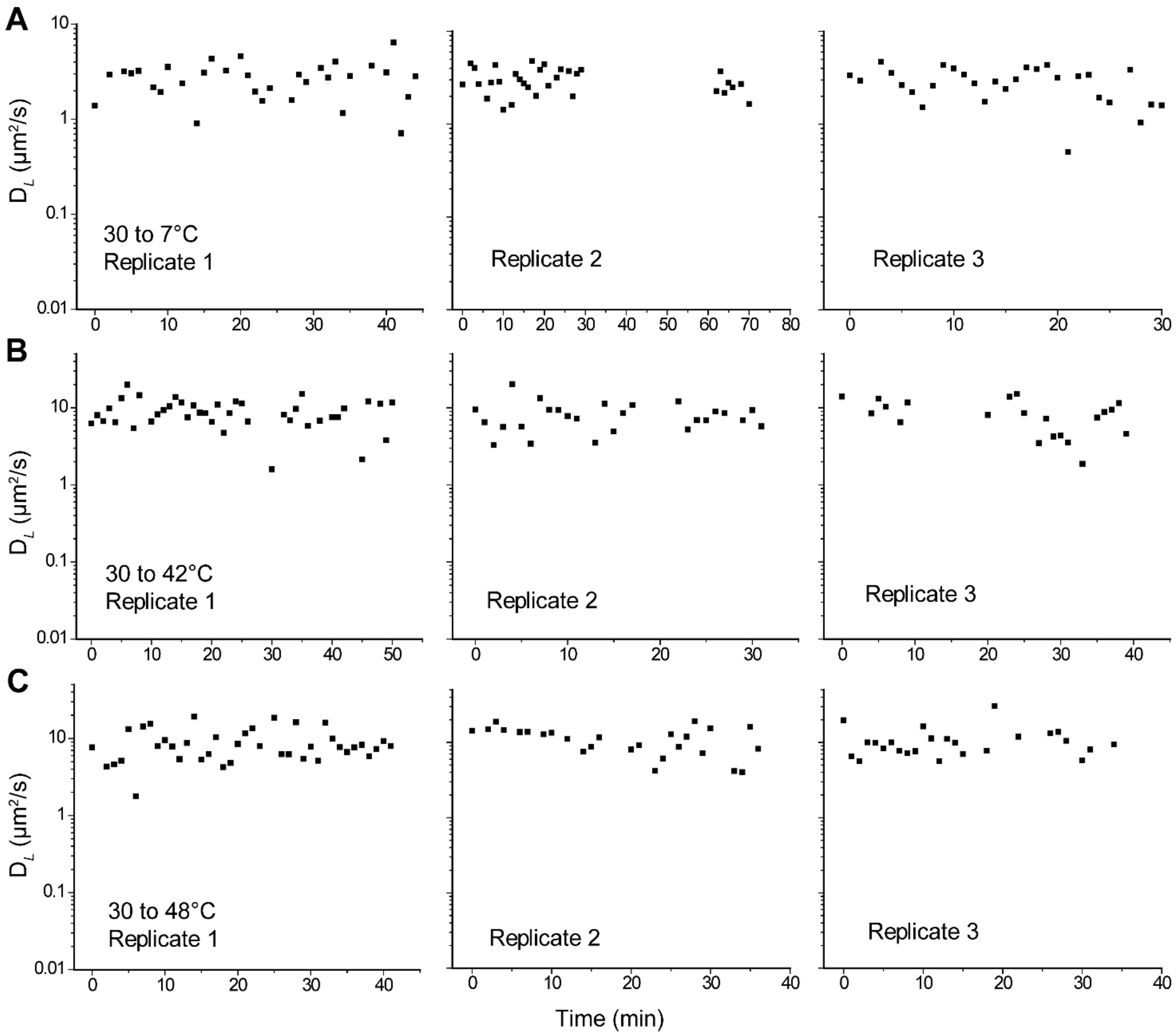

Supplementary Figure 7. Variation in D_L (GFP) $_{\text{plotted}}$ against the measuring time for three replicate samples. (A) Cells shifted from 30 to 7°C; (B) 30 to 42°C and (C) 30 to 48°C. These plots display the D_L values obtained from individual cells against the measuring time.

2.2 Supplementary Tables

Supplementary Table 1: Diffusion coefficients of GFP in *L. monocytogenes* EGD-e grown in different media

	**N total**	**Mean (µm²/s)**	**SD (µm²/s)**	**1st Quartile (Q1) (µm²/s)**	**Median (µm²/s)**	**3rd Quartile (Q3) (µm²/s)**	**Interquartile Range (Q3 - Q1) (µm²/s)**	**Differences between Mean and Median (%)**
In BHI at 30 °C								
Exponential phase	111	5.93	2.43	4.19	5.70	7.20	3.01	4.07
Stationary phase	100	4.39	1.87	3.18	4.37	5.31	2.13	0.41
In CDM at 30 °C								
Exponential phase	125	7.05	2.69	5.27	6.61	8.57	3.30	6.69
Stationary phase	148	4.95	2.21	3.63	4.74	6.07	2.44	4.56
In BHI at 37 °C								
Exponential phase	104	5.52	2.81	3.70	5.58	7.22	3.52	0.96
Stationary phase	101	5.02	3.30	2.93	4.79	6.55	3.62	4.84
In CDM at 37 °C								
Exponential phase	130	5.43	2.61	3.46	5.22	7.13	3.67	4.04
Stationary phase	117	5.80	3.06	3.74	5.42	7.53	3.79	7.05
+25GFP								
In BHI at 30 °C	42	0.45	0.48	0.20	0.38	0.53	0.33	18.17
In CDM at 30 °C	79	1.58	1.25	0.53	1.19	2.41	1.88	32.38

Supplementary Table 2: Diffusion coefficients of -8GFP in *L. monocytogenes* EGD-e as a function of osmotic stress

	N total	Mean (µm²/s)	SD (µm²/s)	1st Quartile (Q1) (µm²/s)	Median (µm²/s)	3rd Quartile (Q3) (µm²/s)	Interquartile Range (Q3 - Q1) (µm²/s)	Differences between Mean and Median (%)
Osmotic adaptation in CDM (Osm) - wild-type								
0.23	125	7.05	2.69	5.27	6.61	8.57	3.30	6.69
0.34	106	5.86	2.65	4.19	5.57	7.05	2.86	5.30
0.61	94	5.11	1.94	3.92	4.80	6.21	2.29	6.61
0.92	100	3.91	2.08	2.53	3.92	5.14	2.62	0.16
1.20	110	3.06	1.67	1.78	2.78	4.22	2.44	9.92
1.38	94	2.83	1.30	1.88	2.88	3.56	1.68	1.56
1.66	85	2.68	1.22	1.82	2.59	3.28	1.46	3.51
1.95	99	1.39	1.27	0.52	1.39	1.78	1.26	0.12
2.37	117	0.86	0.60	0.41	0.79	1.20	0.79	9.64
Osmotic adaptation in CDM (Osm) - *ΔsigB*								
0.23	54	7.80	2.62	6.16	7.94	9.29	3.13	1.71
0.34	32	6.11	1.93	4.56	5.78	7.22	2.67	5.63
0.61	35	5.87	2.28	4.44	5.68	7.20	2.76	3.34
0.92	31	4.80	2.31	2.73	4.47	6.24	3.51	7.38
1.20	47	4.86	1.72	3.67	4.72	5.42	1.75	2.87
1.38	41	4.07	1.54	3.22	3.97	4.51	1.29	2.41
1.66	40	3.01	1.08	2.22	3.04	3.45	1.23	0.89
1.95	46	3.28	1.24	2.37	3.14	3.89	1.52	4.48
2.37	51	3.56	1.44	2.60	3.50	4.50	1.90	1.60
Osmotic shock in CDM (Osm) - wild-type								
0.23	125	7.05	2.69	5.27	6.61	8.57	3.30	6.69
0.61	95	5.02	2.55	3.53	4.62	6.17	2.64	8.71
1.20	101	3.82	1.67	2.82	3.64	4.60	1.78	4.86
1.66	88	2.78	1.64	1.87	2.58	3.38	1.52	7.90

Supplementary Table 3: Diffusion coefficients of -8GFP in *L. monocytogenes* EGD-e as a function of temperature

	N total	Mean (1) (µm²/s)	SD (µm²/s)	1st Quartile (Q1) (µm²/s)	Median (µm²/s)	3rd Quartile (Q3) (µm²/s)	Interquartile Range (Q3 - Q1) (µm²/s)	Differences between Mean and Median (%)
Growth in BHI								
7 °C	93	2.87[c]	1.35	2.01	2.78	3.50	1.49	3.21
20 °C	106	4.20[b]	1.48	3.17	3.96	5.21	2.04	6.08
30 °C	111	5.93[a]	2.43	4.19	5.70	7.20	3.01	4.07
37 °C	104	5.52[a]	2.81	3.70	5.58	7.22	3.52	0.96
42 °C	32	3.30[bc]	3.38	0.64	2.01	4.73	4.08	64.69
Growth in CDM								
7 °C	112	2.04[d]	0.96	1.45	1.87	2.39	0.94	9.18
20 °C	188	3.28[c]	2.23	1.67	2.76	4.08	2.41	19.04
30 °C	125	7.05[a]	2.69	5.27	6.61	8.57	3.30	6.69
37 °C	130	5.43[b]	2.61	3.46	5.22	7.13	3.67	4.04
Temperature shift in CDM								
7 °C	93	2.87[c]	1.05	2.12	2.86	3.54	1.42	0.37
30 °C	125	7.05[a]	2.69	5.27	6.61	8.57	3.30	6.69
42 °C	88	8.57[b]	3.63	6.49	8.34	10.90	4.42	2.80
48 °C	91	9.95[b]	4.63	6.67	8.70	13.20	6.53	14.36

(1) Mean values indicated by different letters (a, b, c, and d) in each experiment are statistically significant difference, one-way ANOVA ($p<0.05$) and Tukey *post hoc* tests ($p<0.01$).
